# Mechanisms of tissue degeneration mediated by periostin in spinal degenerative diseases and their implications for pathology and diagnosis: a review

**DOI:** 10.3389/fmed.2023.1276900

**Published:** 2023-10-31

**Authors:** Tomohito Yoshihara, Tadatsugu Morimoto, Hirohito Hirata, Masatoshi Murayama, Toshihiro Nonaka, Masatsugu Tsukamoto, Yu Toda, Takaomi Kobayashi, Kenji Izuhara, Masaaki Mawatari

**Affiliations:** ^1^Department of Orthopaedic Surgery, Faculty of Medicine, Saga University, Saga, Japan; ^2^Division of Medical Biochemistry, Department of Biomolecular Sciences, Saga Medical School, Saga, Japan

**Keywords:** periostin, pathway, biomarker, mechanical stress, inflammation, spinal degenerative diseases, bone metabolic diseases

## Abstract

Periostin (POSTN) serves a dual role as both a matricellular protein and an extracellular matrix (ECM) protein and is widely expressed in various tissues and cells. As an ECM protein, POSTN binds to integrin receptors, transduces signals to cells, enabling cell activation. POSTN has been linked with various diseases, including atopic dermatitis, asthma, and the progression of multiple cancers. Recently, its association with orthopedic diseases, such as osteoporosis, osteoarthritis resulting from cartilage destruction, degenerative diseases of the intervertebral disks, and ligament degenerative diseases, has also become apparent. Furthermore, POSTN has been shown to be a valuable biomarker for understanding the pathophysiology of orthopedic diseases. In addition to serum POSTN, synovial fluid POSTN in joints has been reported to be useful as a biomarker. Risk factors for spinal degenerative diseases include aging, mechanical stress, trauma, genetic predisposition, obesity, and metabolic syndrome, but the cause of spinal degenerative diseases (SDDs) remains unclear. Studies on the pathophysiological effects of POSTN may significantly contribute toward the diagnosis and treatment of spinal degenerative diseases. Therefore, in this review, we aim to examine the mechanisms of tissue degeneration caused by mechanical and inflammatory stresses in the bones, cartilage, intervertebral disks, and ligaments, which are crucial components of the spine, with a focus on POSTN.

## 1. Introduction

The prevalence of spinal degenerative diseases (SDDs) is on the rise with the growing aging population. SDD-induced low back pain and neurological disorders can impact patients’ quality of life (QOL), work productivity, and impose a substantial economic burden on individuals, their families, and the society (1). The risk factors for SDDs include aging, heavy physical labor involving mechanical stress, trauma, genetic predisposition, obesity, and metabolic syndrome (2–4). Although the causes of SDDs remain unclear, understanding the etiologies will be critical for its prevention and treatment. SDD is also a broad term encompassing various diseases resulting from the degeneration of the spinal structure. Furthermore, the combination of spinal components and their degenerative diseases include osteoporosis, facet joint osteoarthritis, disk degeneration, and lumbar spinal stenosis (LSS) in the bones, cartilage, intervertebral disks (IVDs), and ligaments, respectively.

**Figure 1:**
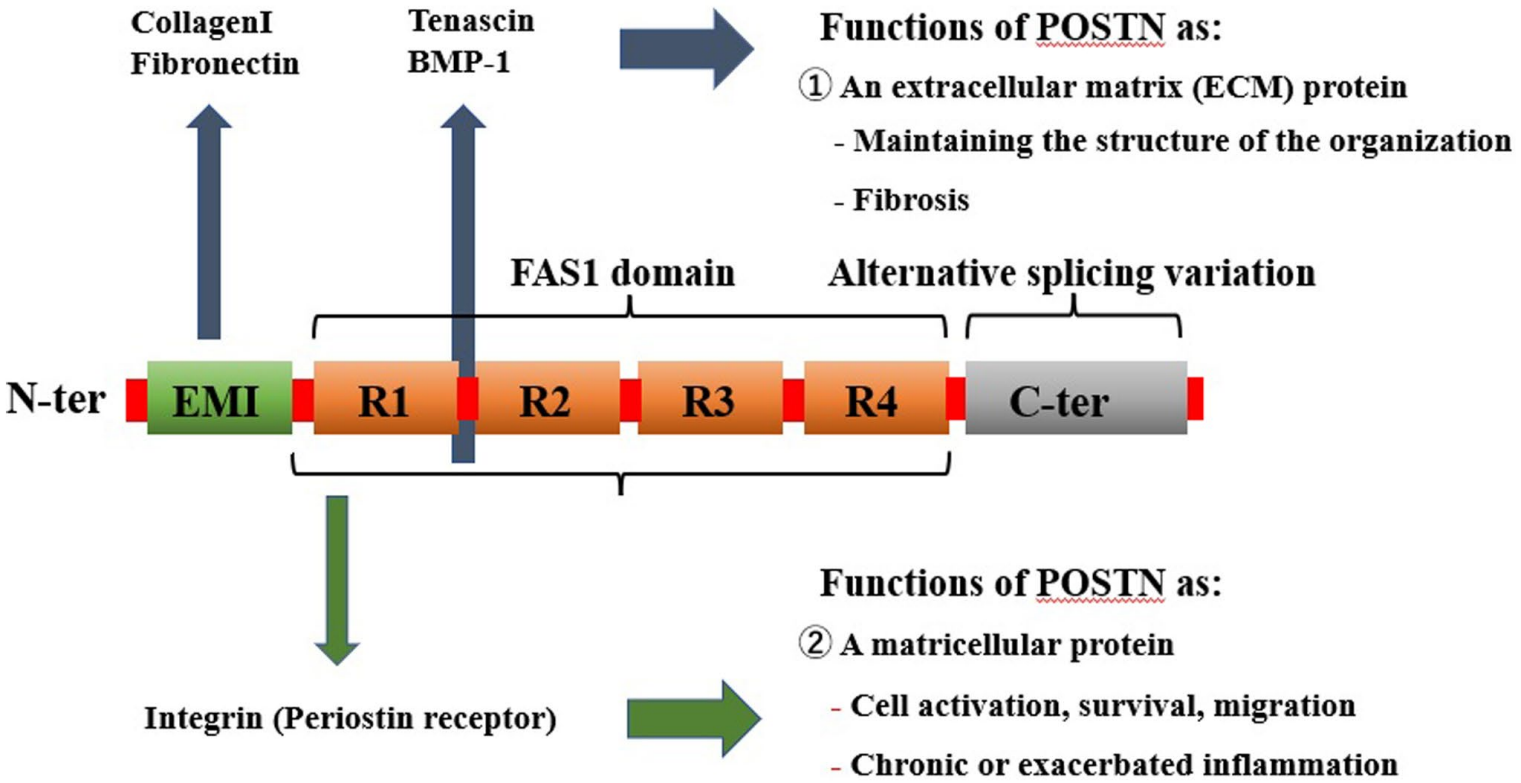
Pathological expression of POSTN as a result of mechanical stress and inflammation systemically causes various diseases in tissues. POSTN is involved in numerous diseases, including orthopedic diseases such as those involving the bone, as well as those affecting the brain, eyes, nasal cavity, lungs, heart, intestine, kidney, skin, and various cancers. POSTN, periostin.

The principal cells of the spinal components include osteocytes, osteoblasts, and osteoclasts in the bone, chondrocytes in the cartilage, medullary nucleus cells in the IVD, and fibroblasts in the ligaments. The progressive degeneration of spinal structures has been attributed to biomechanical injury or stress and biochemical stressors that can adversely affect the regular activity of cells and tissues in the spinal components. Furthermore, these risk factors, either independently or in combination, contribute to the complex interplay between mechanical and biochemical factors leading to the etiology of SDDs. However, it is primarily mechanical stress and inflammation that direct degeneration of the spinal components. Periostin (POSTN) is a type of extracellular matrix (ECM) protein closely associated with mechanical stress, inflammation, and aging, and it has been reportedly linked with the onset and progression of SDDs. Originally, POSTN was named based on its expression in the periosteum of long bones (5). It is primarily expressed in collagen-rich adult connective tissues, such as heart valves, skin, periodontal ligaments, tendons, and bone, which are under constant mechanical stress, predominantly from the ontogenetic stage onwards (5, 6). POSTN is also upregulated during fracture healing, indicating that it may play an important role in bone maintenance and regeneration (7, 8). Additionally, POSTN is expressed in the musculoskeletal cells that structure the spine, including bone marrow-derived mesenchymal stem cells (MSCs) (9–11), osteocytes and osteoblasts (7, 8, 12–18), chondrocytes (10, 11, 19–22), nucleated cells (23–25), and fibroblasts (19, 26, 27) because of its characteristic response to mechanical stress. Notably, POSTN has been expressed in and has influenced the differentiation of these cells. Thus, the characteristic response of POSTN to mechanical stress highlights its role in tissue repair and injury healing (28). However, its overexpression has been observed in various diseases, which are characterized by inflammation, fibrosis, atherosclerosis, and tumorigenesis. POSTN exerts different roles in tissue development and disease progression, including brain injury, ocular diseases, chronic rhinosinusitis, allergic rhinitis, dental diseases, atopic dermatitis, scleroderma, eosinophilic esophagitis, asthma, cardiac diseases, lung diseases, liver diseases, chronic kidney diseases, inflammatory bowel disease, and osteoarthrosis (29) (Figure 1). POSTN has been associated with several chronic inflammatory diseases owing to its interaction with inflammatory cytokines (26, 27, 30). Although POSTN has been reportedly involved in the onset and progression of inflammatory diseases by activating the transforming growth factor β (TGF-β), phosphoinositide-3 kinase/protein kinase B (PI3K/Akt), Wnt, Ras homolog family member A/Rho-associated coiled-coil containing protein kinase (RhoA/ROCK), nuclear factor kappa B (NF-κB), mitogen-activated protein kinase (MAPK), and Janus kinase (JAK) pathways, these pathways have also been linked with SDD onset and progression (31). This suggests that crosstalk exists between bone metabolism and the immune system, which explains the link between the regulation of bone metabolism and the mechanisms that trigger inflammation in allergic diseases (32). Additionally, chronic low-level inflammation is involved in the development and progression of osteoporosis (33), disk degeneration (34), ankylosing spondylitis (AS) (35, 36), and ossification of the posterior longitudinal ligament (OPLL) (37, 38).

Metabolic syndrome is one of the risk factors for SDDs and is strongly associated with lifestyle (39). Metabolic syndrome is triggered by visceral fat, which leads to lipid abnormalities, hyperglycemia, and hypertension (39). Additionally, metabolic syndrome induces “meta-inflammation,” a persistent, low-grade systemic inflammation caused by metabolic stress, which places biochemical stress on systemic tissues (40).

POSTN and metabolic syndrome are characterized by inflammation, and an association between POSTN and metabolic syndrome has also been identified (40, 41). Recently, a novel perspective regarding the aging process has emerged, centered around cellular senescence (42–44). Cellular senescence results in senescent cells (SCs) exhibiting age-dependent accumulation (45, 46). SCs also produce a senescence-associated secretory phenotype (SASP) that secretes various inflammatory cytokines; osteoporosis, sarcopenia, intervertebral disk degeneration (IVDD), and osteoarthritis have been reported as SASP-associated spinal-musculoskeletal diseases (42–44). POSTN is also involved in the senescence of osteoblasts, chondrocytes, medullary nucleus cells, and fibroblasts and in SDD development and progression through SASP. Osteoporosis, sarcopenia, IVDD, and osteoarthritis have been reported as musculoskeletal diseases associated with SASP (46). This age-related inflammation is mediated by NF-κB factors (47), regulated by polymorphisms in various immune system genes and has variable rates of association with chronic inflammatory diseases such as Alzheimer’s disease (48). Additionally, POSTN has been implicated in the aging of osteoblasts, chondrocytes, medullary nucleus cells, and fibroblasts via the NF-κB pathway, which can be inferred to be involved in the development and progression of SDDs (49–52). Furthermore, POSTN is strongly associated with various aspects of development, metabolism, and immunity, and its expression is linked with the degeneration of spinal constructs, thereby playing an important role in the differentiation of associated cells and the activation and progression of pathological conditions.

Zhu et al. (53) reviewed the relationship between SDD and POSTN, mainly in relation to bone metabolism and IVDD. SDDs include intervertebral joint degeneration due to cartilage degeneration and lumbar spinal canal stenosis due to ligamentous degeneration. For a more comprehensive understanding of the relationship between POSTN and SDD, the relationship between POSTN and intervertebral joint degeneration and lumbar spinal canal stenosis should also be included.

Section 2 outlines the structure and function of POSTN. We also review the POSTN signaling pathway and examine its relationship to cells of spinal components (bone, cartilage, disk, and ligaments) given that mechanotransduction—which involves the conversion of mechanical stress into biochemical signals and intracellular changes such as activation of signaling pathways—is essential in development, physiology, and pathology. Section 3 compiles the findings that highlight the usefulness of POSTN as a biomarker for serum, tear, joint fluid, sputum, and urine samples. Notably, large-scale epidemiological studies have accumulated valuable data on serum POSTN levels. POSTN has been intensively studied and clinically used as a biomarker, particularly for inflammatory and allergic diseases. The subsections introduce basic topics related to the biological roles of POSTN in bone marrow-derived MSCs, osteoblasts, osteoclasts, chondrocytes, and annulus fibrosus and medullary nucleus cells, which are closely related to SDDs. Section 4 focuses on the relationship between POSTN and the bone, particularly osteoporosis and vertebral fractures; section 5 highlights the correlation between POSTN and cartilage, specifically its relevance to osteoarthritis. Section 6 focuses on IVDs, encompassing disk degeneration and AS, and section 7 focuses on the relationship between POSTN and ligaments, with a focus on lumbar spinal canal stenosis. Therefore, we propose that a better understanding of the function of POSTN in SDD etiology could lead to the development of treatments for these diseases. Furthermore, this review summarizes the existing evidence linking POSTN to various SDDs, outlining the nature and role of POSTN as well as the POSTN abnormalities associated with the degeneration of spinal components.

## 2. Structure and function of POSTN

### 2.1. Structure of POSTN

**Figure 2:**
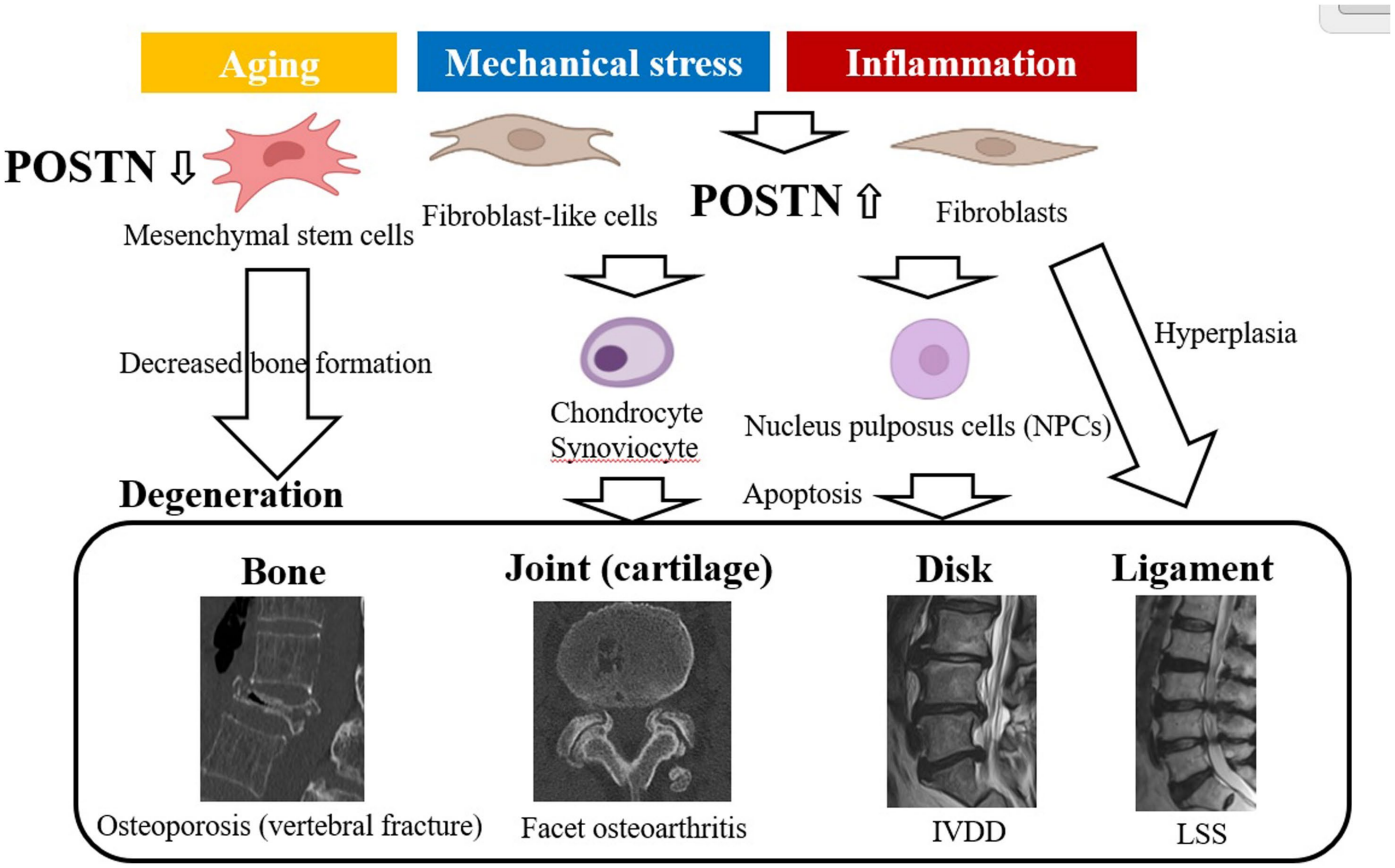
Structure of POSTN as an ECM protein as a conventional ECM protein, POSTN maintains tissue and organ structure or generates fibrosis, whereas as a matricellular protein, it is involved in cell activation. POSTN comprises an EMI domain at the N terminus, four tandemly aligned FAS1 domains in the middle, and splicing domains at the C terminus. ECM proteins or a proteinase that can bind to the EMI domain or the tandem repeat of four FAS1 domains are depicted. In contrast, POSTN binds to integrin molecules on the cell surface, transducing intracellular signals. POSTN, periostin; ECM, extracellular matrix; FAS1, fasciclin 1.

POSTN is a 90 kDa ECM protein, and it consists of 836 amino acids in humans. It was originally named osteoblast-specific factor 2 (OSF-2) when initially cloned from a cDNA library prepared from the mouse osteoblastic cell line MC3T3-E1 (5, 16). The POSTN protein structure comprises an N-terminal EMI domain, a carboxy-terminal domain (CTD), and a tandem repeat of four fasciclin 1 (FAS1) domains (54–56) (Figure 2).

These components function in the following ways: first, the N-terminal EMI domain confers the capacity to interact with collagen I and fibronectin. Second, CTD is a site of proteolytic cleavage that generates the different isoforms of POSTN. Third, the intermediate region, which comprises four FAS1 domains, including binding sites for bone morphogenetic protein 1 (BMP-1), αVβ3, and αVβ5 integrins, bestows the ability to mediate cell adhesion and migration.

In rats, mice, and humans, exons 17–22 are symmetrical and have similar lengths. Furthermore, these exons share remarkable sequence similarity at the DNA level and are alternatively spliced. Various combinations of three of these six exons depict eight alternative splicing variants resulting in eight protein-coding isoforms, and there is yet another noncoding ninth isoform. Moreover, POSTN is frequently overexpressed in some cancers, and its various isoforms are generally believed to exhibit tissue-specific expression (19).

### 2.2. Function of POSTN

POSTN is characterized as both a matricellular protein and an ECM protein belonging to the fasciclin family. An N-terminal EMI domain, a CTD, and a tandem repeat of four FAS1 domains have been demonstrated to bind to several proteins, including ECM proteins, and exhibit different biological functions. Specifically, the N-terminal EMI domain and tandem repeats of the four FAS1 domains bind to ECM proteins, such as type 1 collagen, fibronectin, tenascin C, and BMP-1, which are involved in the structural maintenance of tissue and fibrosis (57, 58).

Numerous reports have shown that POSTN also binds to integrins, such as αVβ1, αVβ3, αVβ5, α6β4, and αMβ2 and activates TGF-β, PI3K/Akt, Wnt, RhoA/ROCK, NF-κB, MAPK, and JAK pathways via these receptors to promote the development and pathogenesis of multiple diseases (Table 1).

**Table 1:** POSTN activates different signaling pathways that are involved in diverse diseases.

Spinal structures	Cells	Interacting pathway	Diseases	Function	Reference
Bone	Osteoblasts	TGF-β pathway	Osteoporosis	Induce differentiation, survival, and apoptosis	(16)
		NF-κB pathway	Osteoporosis	Induce expression of Runx2 and effects on osteoblast differentiation	(17)
		Wnt/β-catenin pathway	Osteoporosis	Induce bone formation	(59)
		PERK pathway	Osteoporosis	Inhibit melatonin-induced cell apoptosis	(18)
	Bone-marrow stem cells	Wnt/β-catenin pathway	Osteoporosis	Mediate osteogenic differentiation of BMSCs to reduce osteoporosis	(10)
Joint	Chondrocytes	PI3K/Akt pathway	Osteoarthritis	Induce collagen and proteoglycan degradation in the cartilage	(21)
		NF-κB pathway	Osteoarthritis	Upregulation of inflammatory cytokines and MMPs	(20)
		Wnt/β-catenin pathway	Osteoarthritis	Induce MMP13 and ADAMTS4 expression, accelerate the pathogenesis of OA	(60)
				Induce collagen and proteoglycan degradation in the cartilage	(21)
		MAPK pathway	Osteoarthritis	Interaction of integrins with matrix proteins activates MAPKs and increases NF-κB signaling	(61)
Disk	Nucleus pulposus cells	NF-κB pathway	IVDD	Accelerates senescence of nucleus pulposus cells	(52)
		Wnt/β-catenin pathway	IVDD	Promotes apoptosis and exacerbates degeneration of nucleus pulposus cells	(62)
		JAK/STAT3 pathway	IVDD	Exacerbates degeneration of nucleus pulposus cells	(63)
Ligament	Fibroblasts	NF-κB pathway	LSS	Upregulation of inflammatory cytokines and MMPs	(64)

By binding to integrins, POSTN converts mechanical and inflammatory stress into biochemical signals, resulting in intracellular changes, such as the activation of various signaling pathways that affect the cells of spinal constructs (bone, cartilage and IVDs, and ligaments, among others). Mechanical stress reportedly induces the binding of POSTN to integrins and interleukin (IL)-6 and matrix metalloproteinases (MMPs) via the FAK-NF-κB pathway, leading to cartilage (50, 64) and ligament (65) degeneration. Inflammatory stress has also been reported to induce POSTN by IL-4, IL-13, and TGF-β (66) and progress cartilage degeneration via the integrin-mediated FAK-NF-κB (20) and Wnt5a-CaMKII pathways (62). However, other reports indicate that POSTN binds to integrin and induces apoptosis via the Wnt/β-catenin (67) and αvβ3 integrin/ERK pathways (68), and the NF-κB pathway (52), causing disk degeneration. Additionally POSTN induces fibroblasts into failing muscles via the FAK/Akt pathway (69) and is regulated by TGF-β (59), resulting in muscle degeneration. POSTN is also expressed in bone cells and osteoblasts, regulating osteoblast and bone formation (8, 70) and is associated with osteoporosis via the sclerostin-mediated Wnt/β-catenin pathway (21, 70–72).

### 2.3. Pathological expression of POSTN resulting from mechanical stress and inflammation causes systemic diseases in tissues

#### 2.3.1. POSTN expression in response to mechanical stress and inflammation

**Figure 3:**
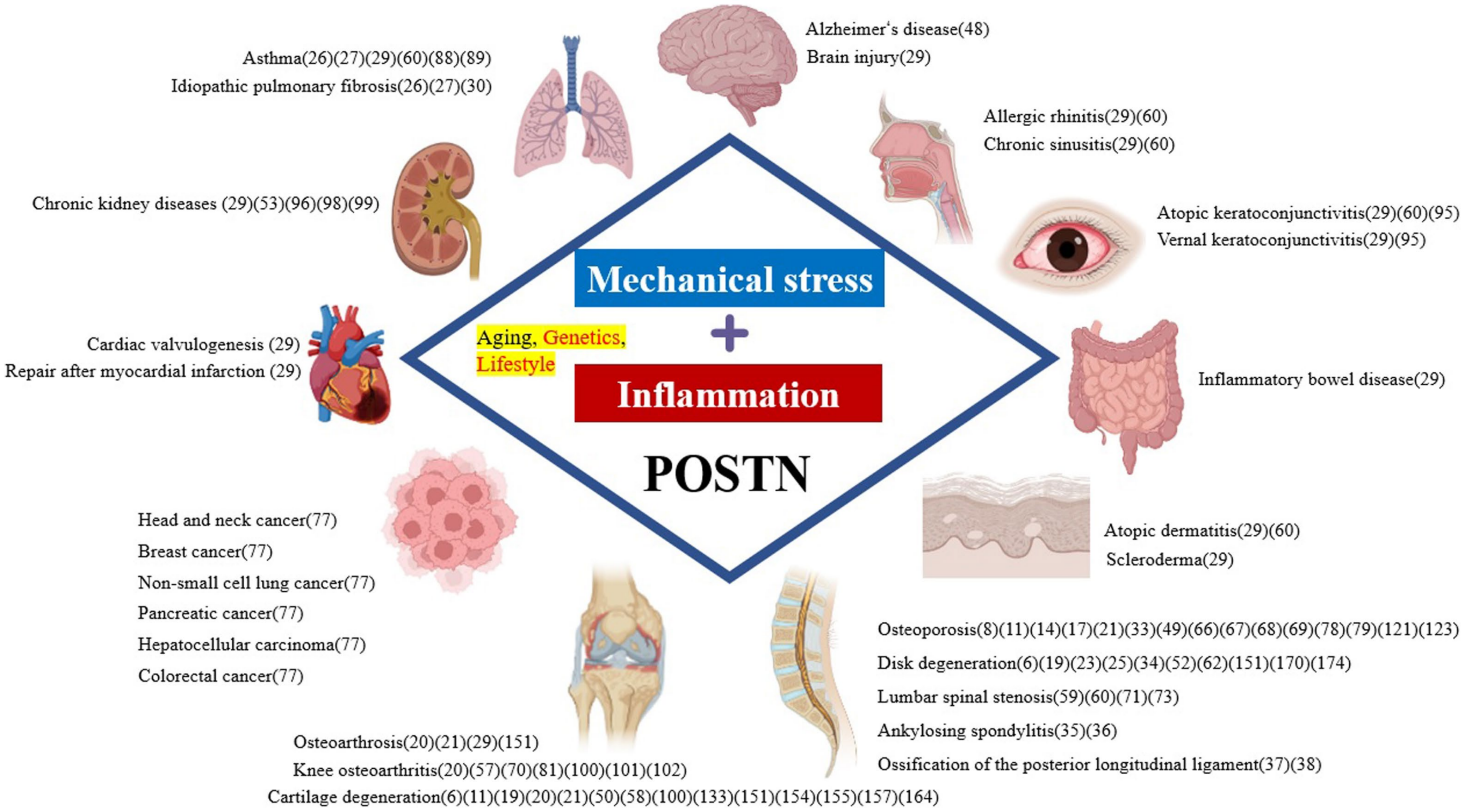
Pathological expression of POSTN resulting from mechanical stress and inflammation causes spinal degenerative diseases. IVDD, intervertebral disk degeneration, LSS, lumbar spinal stenosis; POSTN, periostin.

POSTN contributes to the development of various disease in different tissues throughout the body and is pathologically expressed primarily by fibroblasts and osteoblasts as a result of mechanical stress and inflammation, aging, and genetic factors (Figure 1). For SDDs primarily caused by mechanical stress and inflammation, POSTN may be pathologically expressed by osteoblasts in the bone, fibroblast-like cells of the annulus fibrous in the joint and IVDs, and fibroblasts in the yellow ligament (19, 20, 65, 67, 73–75) (Figure 3).

POSTN expression decreases in MSCs with aging, reducing bone formation (73). In the joints, POSTN promotes the expression of inflammatory cytokines and MMPs in chondrocytes and synoviocytes, causing apoptosis, which leads to osteoarthritis (20, 74).

Fibroblast-like cells increase POSTN expression and activate the Wnt/β-catenin pathway in IVD, exacerbating apoptosis and degeneration in nucleus pulposus (NP) cells (NPCs) (19, 67). However, POSTN expression is upregulated in fibroblasts, which is the main component of ligamentum flavum (LF) exposed to TGF-β1 induction by mechanical stress, and the induction of IL-6 via the NF-κB (65) and JAK1/STAT3 pathways causes inflammation and LF thickening (75).

#### 2.3.2. Cellular aging and POSTN

Recently, a cellular senescence-centric view of the aging process has emerged. Notably, in baboon skin, cellular senescence has been identified as a possible contributing factor to chronic inflammation in older individuals (45). The surviving senescent cells (SC) produce SASPs, which secrete various inflammatory cytokines, promoting chronic inflammation and carcinogenesis. Therefore, the accumulation of SC load with aging destroys tissue structure and function and is an important factor in increasing disease risk and mortality in older individuals (43). This age-related inflammation is mediated by NF-κB factors (47), regulated by polymorphisms in various immune system genes, and has variable rates of association with chronic inflammatory diseases such as Alzheimer’s disease (48). POSTN has been implicated in the aging of osteoblasts (49), chondrocytes (21), medullary nucleus cells (52), and fibroblasts (76) via the NF-κB pathway, which can be inferred to be involved in SDD development and progression through SASP. POSTN expression-mediated musculoskeletal diseases associated with SASP include osteoporosis (49), osteoarthritis (21), IVDD (52), and LSS (77). Furthermore, the siRNA-mediated knockdown of *POSTN* encoding the protein POSTN and the inactivation of POSTN by neutralizing antibodies have been documented to alleviate aging in IVDD (52). Therefore, POSTN-neutralizing antibodies are a therapeutic agent for spinal musculoskeletal diseases owing to their anti-aging properties.

## 3. Characteristics of POSTN as a biomarker

POSTN is readily transferred or secreted from inflammatory sites into various body fluids. Consequently, its levels are reportedly increased in the blood, urine, sputum, exhaled breath condensate, and tears in various conditions. Therefore, the POSTN level in body fluids may serve as a more direct biomarker for local inflammation than that in the serum (66). However, it is difficult to accurately detect increased inflammatory sites with serum concentrations when they are very high or very low, as in the cases of ECM proteins like fibronectin and vitronectin (~100 μg/mL) or cytokines (~10 pg./mL), respectively. Conversely, the concentration of serum POSTN is approximately 10 ng/mL, which is adequate to accurately detect an increase at the site of inflammation. A few drawbacks exist when serum POSTN is used as a biomarker. First, the basal level of serum POSTN in childhood is high and varies with age (78). Second, the isoforms of POSTN and immunoglobulin A form a complex in the serum, which may affect serum POSTN measurements (79). Another challenge in assessing POSTN levels is the variations in absolute values determined using different POSTN assay kits, and standardization is necessary for reliable assessment of POSTN values. Baseline values of serum POSTN may not be significantly associated with spinal musculoskeletal disease. POSTN was initially identified in osteoblast cell lines (5), and its expression was prominent in the periosteum, indicating its involvement in bone tissue development and function (7). As described above, POSTN plays crucial roles in components of the spinal musculoskeletal system, such as the bone, cartilage, IVDs, and ligaments, and is closely related to the pathogenesis of diseases affecting these components. Nevertheless, the clinical use of POSTN as a biomarker for diagnosis and severity determination, and the use of neutralizing antibodies as therapeutic agents has been explored for allergic inflammatory diseases (80), and such investigations have been limited for SDDs.

### 3.1. Serum POSTN

POSTN is a matricellular protein that plays a vital role in allergic disease development. It is a downstream molecule of IL-4 and IL-13 that is involved in the pathogenesis of fibrosis and has been established as a serum biomarker of remodeling in various allergic diseases, including asthma, allergic rhinitis, chronic sinusitis, atopic dermatitis, and allergic conjunctivitis in adults (66). Regarding malignancy, elevated POSTN levels have been detected in the serum of patients with cancer, suggesting its usefulness as a biomarker for diagnosis, metastasis, and prognosis (81). Malignancies associated with elevated serum POSTN levels include head and neck cancer, breast cancer, non-small cell lung cancer, hepatocellular carcinoma, pancreatic cancer, and colorectal cancer. POSTN has been reported as a potential biomarker for osteoporosis, fractures, knee osteoarthritis, AS, and OPLL in the musculoskeletal system. Additionally, it is produced by osteoblasts and osteocytes (7, 16), is involved in osteoblast proliferation, differentiation, survival, and collagen mineralization, and is strongly associated with osteoporosis (82). Higher serum POSTN levels have been reportedly associated with lower bone mineral density and observed in women with acute osteoporotic hip fractures (83). Increased serum POSTN levels in hip fractures in older Chinese women are also reported during the early healing phase, suggesting that POSTN plays a role in bone repair (84). Additionally, increased serum POSTN levels have been noted in patients with AS with increased disease activity and systemic inflammation (35) and in advanced groups of OPLL (37), and the levels positively correlated with the radiological severity of knee osteoarthritis (85). Therefore, considering the characteristics of POSTN in clinical disease, several researchers have proposed POSTN as a biomarker for diagnosing osteoporosis, assessing fracture repair status, and predicting knee osteoarthritis severity (58, 86).

No difference has been found in POSTN levels in children by sex (87, 88). Although some reports on sex-based differences in POSTN levels in adults were insignificant (88), others have shown higher POSTN levels in women (89). Moreover, as osteocytes and periosteal osteoblasts produce POSTN, serum POSTN levels are greatly influenced by the rate of bone metabolism during childhood. Therefore, they are significantly high in infants, decline until the age of 7 years, and increase until the age of 15 years during their growth period (adolescence) (50, 78). Basal serum POSTN levels exceed 100 ng/mL during childhood and adolescence, and serum POSTN has been reported to decrease to ~50 ng/mL once bone growth ceases (90). It has also been reported to be constant and not age-dependent after adulthood (88, 89). Caswell-Smith et al. (88) reported that normal reference range values were observed across the age range from 18 to 75 years, regardless of sex, with 90% confidence intervals of 35.0 and 71.1 ng/mL (median 50.1 ng/mL). Furthermore, differences based on ethnicities in serum POSTN levels reportedly tend to be higher in Asians than in Caucasians (88, 89). Serum POSTN levels may also increase with age because chronic inflammation-related diseases, which affect its levels, also increase age-dependently (91). Therefore, when considering ethnic and age differences, investigation of the reference range of serum POSTN levels in large population studies should be the focus of future research.

### 3.2. Sputum POSTN

Although serum POSTN is associated with type 2 inflammation in the airways of patients with asthma (92, 93), systemic POSTN levels are derived from multiple sources, including age, body mass index, and bone formation. As serum POSTN levels reflect systemic POSTN levels (94, 95) and the utility of POSTN derived from the diseased organ requires further investigation. However, information on the use of sputum POSTN as a biomarker in asthma is limited because the detection levels are low with the currently available POSTN assays (96). Ono et al. (96) presented an enzyme-linked immunosorbent assay to improve the analysis of sputum POSTN by detecting the cleavage product of the POSTN protein. Notably, sputum POSTN showed associations with blood and sputum eosinophils. Furthermore, sputum POSTN, rather than serum POSTN, correlated with reduced lung function and sputum IL-13 and was decreased by oral corticosteroid treatment (96).

### 3.3. Tear POSTN

Atopic keratoconjunctivitis (AKC) and vernal keratoconjunctivitis (VKC) are chronic forms of allergic conjunctivitis associated with corneal changes, such as corneal ulceration and formation of giant papillae, tissue remodeling, and fibrosis, which leads to vision loss; therefore, accurate diagnosis and appropriate treatment are important (97, 98). Consequently, the development of novel biomarkers to accurately estimate disease severity is necessary (27). POSTN produced in the conjunctival tissues stimulated by IL-13 may contribute to the pathogenesis of ocular allergic diseases. Furthermore, tear POSTN can be potentially applied as a biomarker to diagnose conjunctivitis in patients with allergies and to evaluate disease severity and effectiveness of treatments in AKC. A study reported that tears from patients with ocular allergic disease had significantly higher POSTN levels than tears from patients with allergy without conjunctivitis or those with AKC, VKC, or seasonal allergic conjunctivitis. Moreover, a trend toward decreased POSTN levels in tears with clinical improvement has been observed in most patients with AKC (99).

### 3.4. Urine POSTN

POSTN has been identified as one of the most represented matricellular proteins in experimental models of kidney diseases. It is commonly involved in inflammation and fibrosis that characterize progressive kidney diseases (100). Currently, the usefulness of established tissue and urine markers to predict outcomes in chronic kidney disease (CKD) is not optimal (101). Therefore, identifying molecules that add value to clinical predictors of treatment response and prognosis in patients with kidney diseases is critical. Given that POSTN is readily secreted from injury sites, and the variations in its humoral levels compared with those in the normal state are easily detectable, its potential role as a biomarker has been proposed (27). Similar to the blood fraction, the urinary fraction of POSTN shares a magnitude of <1 ng/mg creatinine; however, this fraction can significantly increase, and there is evidence that points toward a positive correlation with disease severity in multiple cases of CKD (54, 102, 103). In a study on a small cohort of patients with immunoglobulin A nephropathy and lupus nephritis, Wantanasiri et al. (103) reported a worse kidney function and higher urinary POSTN excretion levels compared with these in healthy controls; additionally, in patients who were responsive to treatment, there was a significant decrease in urinary POSTN levels at 6 months of follow-up. Therefore, urinary POSTN may be a useful biomarker for assessing the extent of renal damage, including CKD.

### 3.5. Synovial fluid POSTN

The expression of the *POSTN* gene and protein and the secretion of POSTN were higher in human osteoarthritis synovial cells than in normal cells, and it increased under the stimulation of the inflammatory cytokine IL-13, suggesting that both POSTN and IL-13 are involved in the pathological development of osteoarthritis (74). However, other studies have shown that POSTN is involved in cartilage matrix degradation and knee osteoarthritis via MMP-13 (104) and MMP-9 (74). Some other studies have reported that the synovial fluid of patients with osteoarthritis had significantly higher concentrations of POSTN than that of patients with rheumatoid arthritis and healthy controls and that these higher levels are positively correlated with the severity and the risk of progression of knee osteoarthritis (85, 105, 106). POSTN levels in synovial fluid positively correlated with the radiological severity of knee osteoarthritis (74, 85). Based on the synovial fluid analysis, POSTN was more highly expressed in subacute anterior cruciate ligament (ACL) injury than in osteoarthritis. POSTN expression was higher in ACL tear remnants and isolated cells and was time-dependent during the period of ACL injury (107). Elevated levels of POSTN after ACL injury may serve as a marker of the repair/healing response, implying a benefit for the joint. Conversely, sustained overexpression of POSTN may contribute to initiating joint degeneration. Moreover, in the latter case, POSTN may be a predictor or target for early intervention to prevent post-traumatic osteoarthritis.

## 4. Role of POSTN in bone metabolism

This section describes the interplay between the ECM component POSTN and the cells involved in bone metabolism.

### 4.1. Cells involved in bone metabolism

The cells involved in bone metabolism are osteoclasts, osteoblasts, and osteocytes. Osteoclasts are multinucleated cells that differentiate from monocyte-macrophage lineage hematopoietic stem cells and are responsible for bone resorption. The differentiation of osteoclasts requires binding the receptor activators of NF-κB ligand (RANKL) expressed by osteoblasts and osteocytes (108). However, osteoblasts differentiate from MSCs and are involved in bone formation by synthesizing, secreting, and mineralizing the bone matrix. Runt-related transcription factor 2 (Runx2) induces the commitment of MSCs to the osteoblast lineage and is necessary for the proliferation and differentiation of osteoblasts (109). Additionally, Runx2 is highly expressed in immature osteoblasts (110). In addition to Runx2, various bone formation signals are involved in bone metabolism, including the Wnt/β-catenin signal. Wnt proteins interact with Frizzled receptors and low-density lipoprotein receptor-related proteins, stabilize β-catenin, and are involved in bone formation (111, 112). Sclerostin is an antagonist of the Wnt/β-catenin pathway and inhibits bone formation. It is primarily produced by osteocytes embedded in the bone matrix and differentiated from osteoblasts. Osteocytes have a long lifespan and can regulate osteoblasts and osteoclasts through various mechanisms, including mechanical stress and aging (113).

Furthermore, it has been suggested that the senescence of osteocytes triggers inflammation through SASP, which disrupts the balance between bone formation and resorption and leads to aging-related bone loss (49, 114). Therefore, understanding the role of POSTN, which is involved in bone metabolism, inflammation, and aging (73, 115), is expected to provide insights into the development of osteoporosis caused by the imbalance in bone metabolism.

### 4.2. Impact of POSTN on the bone

POSTN is expressed in cells involved in the skeleton, such as MSCs and osteoblasts, and functions as a structural molecule of the bone matrix and a signaling molecule that promotes the function of osteoblasts and bone formation through integrin receptors and the Wnt/β-catenin pathway (70). POSTN is primarily secreted by bone marrow MSCs (116) and reportedly promotes the differentiation and proliferation of MSCs into osteoblasts (7, 12). Furthermore, POSTN is regulated by TGF-β, which is involved in bone formation, and plays a role in cell adhesion. Overexpression of POSTN in MC3T3-E1 osteoblasts enhances cell proliferation and differentiation (16). Similarly, its overexpression in rats increases bone formation rate and mass (117). Knockout (KO) of POSTN in MC3T3-E1 cells significantly reduces the expression of Runx2 (17). Furthermore, in the same experiment, the expression of RANKL, which is involved in osteoclast formation, was also significantly reduced, and this is presumed to occur through the NF-κB signaling pathway (17). Although there is a possibility that POSTN may also impact osteoclasts, no reports exist indicating its direct involvement; therefore, further investigation is warranted. In POSTN-KO mice, adhesion of osteoblasts to the bone matrix is impaired, leading to an impact on differentiation toward mature osteoblasts and significantly decreased expression of osteoblast differentiation markers, such as Runx2, osteocalcin, osteopontin, and alkaline phosphatase. Additionally, POSTN-KO mice exhibit reduced bone mass and compromised cortical bone structure, resulting in decreased bone strength (118–120). POSTN also protects osteoblasts from apoptosis by inhibiting the endoplasmic reticulum stress-associated eukaryotic initiation factor 2α (eIF2α)-activating transcription factor 4 (ATF4) pathway through the suppression of the protein kinase R-like endoplasmic reticulum kinase (PERK) pathway (18). Therefore, these findings indicate that POSTN may be involved in the proliferation, differentiation, and anti-apoptotic processes of osteoblasts, potentially influencing bone mass and strength.

### 4.3. POSTN-mediated effect of mechanical stress on the bone

Bone mass is maintained under mechanical stress and decreases when the stress is removed. Signaling molecules, such as prostaglandin E2, nitric oxide (NO), and adenosine triphosphate, have reportedly been involved in bone formation under loading conditions (121–124). Additionally, the Wnt/β-catenin pathway has been suggested to be crucial in bone formation in response to mechanical stress (125). The expression of sclerostin, which inhibits the Wnt/β-catenin pathway, is downregulated for bone formation under loading conditions (126). POSTN is known to increase in response to mechanical stress and has been shown to suppress sclerostin expression, thereby influencing bone formation (14, 127). Specifically, POSTN also plays an essential role in bone formation under loading conditions.

### 4.4. Impact of POSTN on aging, inflammation, and osteoporosis

Recent advances in our understanding of bone remodeling processes support the theory that many inflammatory cytokines are involved in the regulation of osteoblasts and osteoclasts and that inflammation is a major contributor to the etiology of osteoporosis (128). A possible causal relationship between the systemic inflammation seen in elderly and the prevalence of osteoporosis with overall aging has been noted.

As aging occurs, estrogen levels decrease, resulting in an imbalance between bone resorption and formation, leading to osteoporosis (129). The decrease in estrogen levels is known to influence the increased expression of inflammatory cytokines such as TNF-α and IL-6, which promote bone resorption (130). Additionally, sustained inflammatory stimulation with aging has been implicated in osteoporosis (131). POSTN, which is associated with inflammation, has also been shown to affect NF-κB in osteoblasts, suggesting its involvement in osteoporosis (17). Furthermore, with aging, POSTN decreases in bone marrow macrophages and BMSCs, leading to reduced bone repair capacity similar to POSTN-KO mice (73, 132). Based on these findings, there is a possibility that POSTN is also involved in osteoporosis resulting from aging.

There have been reports in several clinical studies indicating that elevated serum levels of POSTN are associated with an increased risk of fractures (133, 134). Additionally, a single nucleotide polymorphism (SNP) rs9603226 in POSTN was found to be unrelated to BMD of the vertebrae but associated with the prevalence of vertebral fractures (135). These studies suggest that POSTN could potentially become a biomarker for predicting fracture risk in the future, suggesting the need for further research.

## 5. Structure and function of cartilage

Facet osteoarthritis is a pathological condition in SDDs caused by cartilage degeneration. Although multiple correlations have been reported between POSTN and lower extremity osteoarthritis, no reports demonstrate an association between facet osteoarthritis and POSTN. Therefore, the relationship between POSTN and lower extremity osteoarthritis will be discussed below as it may be similar to the relationship between facet osteoarthritis and POSTN.

Articular cartilage (AC) is a highly specialized tissue composed of chondrocytes and an ECM comprising types II, IX, and XI collagen and proteoglycans (136), covering and facilitating load distribution between adjacent bones (137). AC has biomechanical properties that combine compressive stiffness, elasticity, and shear resistance, and while appropriate mechanical stress and dynamic loading are important for the normal growth and maintenance of AC to protect, excessive stress can damage the cartilage (138, 139).

Aging (140), heavy labor (mechanical stress) (141, 142), trauma (143), genetic predisposition (144, 145), obesity, and metabolic syndrome (146, 147) have been identified as risk factors for osteoarthritis. In particular, trauma and aging induce mechanical stress, chronic inflammation, and oxidative stress (50, 148), resulting in an imbalance in the regulation of the ECM metabolism of chondrocytes. Obesity and metabolic syndrome appear to be risk factors for osteoarthritis in the load-bearing joints of the lower extremities and non-load-bearing joints (wrist and temporomandibular joints), indicating that systemic chronic low-level inflammation may play a role in its development (146, 147, 149). Therefore, although mechanical and genetic factors have classically been shown to play a major role in osteoarthritis development, many reports indicate that “meta-inflammation,” which is a persistent, low-grade systemic inflammation caused by metabolic stress, plays a vital role in osteoarthritis development and progression (150). Additionally, cartilage tissue is vascular-free and has a slow turnover, making it prone to accumulating senescent cells (SCs) (151). Therefore, SCs actively secrete cytokines, leading to tissue degeneration of the cartilage and, ultimately, osteoarthritis (152).

Molecular mechanisms are currently being investigated for developing osteoarthritis therapies. Inflammatory cytokines such as IL-1, IL-6, IL-8, and TNF-α are involved in cartilage degradation by stimulating MMP expression, which causes matrix destruction (60, 153, 154), and NO has been suggested to play a role in inducing chondrocyte apoptosis through its function as an oxidative stress agent (155). Wnt/β-catenin signaling also plays an important role in the development and function of AC and has recently been implicated in osteoarthritis progression (156). Additionally, gene expression analysis of osteoarthritis cartilage is underway, and a link to POSTN in cartilage metabolism has been highlighted (61).

### 5.1. Impact of POSTN on the cartilage

POSTN is a TGF-β-inducible ECM protein that is expressed in the cartilage (19), bone (157), meniscus (107), and ligament (19, 104) in joints. Normal articular chondrocytes express isoforms 1 and 8 at high levels and exhibit isoforms that differ from ACL progenitor cells, suggesting their splice-specific regulation in chondrocytes (19).

POSTN expression is elevated in osteoarthritis cartilage (20, 61) and also significantly increased in the cartilage of surgically-induced osteoarthritis rats (140). qPCR was used to explore the molecular differences between light and dark hypertrophic chondrocytes, and the most highly aberrantly expressed dark cell-specific gene was *POSTN* (158). Furthermore, POSTN expression was elevated in the synovial fluid of patients with osteoarthritis with the progression of osteoarthritis grade (159), suggesting that it is associated with osteoarthritis. Moreover, many other studies have elucidated the role and mechanism of POSTN in osteoarthritis. Chijimatsu et al. (20) showed that the application of POSTN to isolated human chondrocytes induced high expression of IL-6 and IL-8 accompanied by the elevated expression of MMP-1, MMP-3, MMP-13, and nitric oxide synthase-2 (NOS2) through NF-κB activation mechanism. POSTN also induced the expression of MMP-13 and a disintegrin and metalloproteinase with thrombospondin motifs 4 (ADAMTS4) in a dose-and time-dependent (61) manner, and it promoted cartilage degeneration via a Wnt/β-catenin/MMP-13/ADAMTS4-or discoidin domain receptor-1 (DDR1)/Akt/Wnt/β-catenin/MMP-13-dependent mechanism (21). Furthermore, in osteoarthritis synovial cells, IL-13 activated signal transducer and activator of transcription 6 (STAT6) to upregulate POSTN expression. POSTN expression was suppressed by dexamethasone, leflunomide (which inhibits STAT6), and hydrobromide (which inhibits Wnt/β-catenin signaling) (61, 74, 159). Therefore, these results indicate that POSTN is a catabolic factor that induces the degradation of collagen and proteoglycans, and it may be a new therapeutic target to prevent osteoarthritis progression (53).

### 5.2. POSTN-mediated effects of mechanical stress on the cartilage

Spinal degeneration includes bony vertebral degeneration and IVDD, which are accelerated by the physiological processes of aging (160) and mechanical pressure (148). In osteoarthritis, mechanical stress is believed to be the primary etiological factor (141, 142), and when disk degeneration occurs, it is expected to result in increased pressure in the intervertebral joints and cartilage degeneration.

POSTN has been expressed in mechanically stressed connective tissue in adults (8, 161). It also suggests that stress and pressure overload induce the expression of POSTN and that IVD overuse and damage are associated with elevated expression (162). Mechanical stress activates the mammalian target of rapamycin (mTOR) signaling and increases the expression of both POSTN and mTOR, resulting in POSTN further enhancing mTOR signaling (161).

In the cartilage, POSTN contributes to the shape retention of biodegradable polymer scaffolds by increasing the mechanical strength of the surrounding fiber tissue comprising POSTN-mediated collagen structures (163). In POSTN-null mice, collagen cross-linking was defective, and resistance to mechanical stress was reduced (6).

According to Chijimatsu et al. (20), the cartilage of the medial tibial plateau in medial osteoarthritis was the site of greatest degeneration by mechanical pressure and had the highest POSTN mRNA levels compared with the femoral head and other areas of osteoarthritis cartilage. Moreover, POSTN was expressed in chondrocytes and the surrounding matrix in the erosive superficial layers, particularly near the fissures of degenerated cartilage; however, it was rarely detectable in the deeper layers. Therefore, this suggests that POSTN may be upregulated in chondrocytes in response to mechanical stress. Furthermore, Nakamura et al. (50) demonstrated that POSTN upregulated the expression of catabolic factors, IL-6 and MMP-3, through integrin αVβ3, focal adhesion kinase, Src, and NF-κB signaling in swaddling hip dislocation models.

### 5.3. POSTN-mediated effects of low-level inflammation on the cartilage

Osteoarthritis is recognized as a systemic and localized state of low inflammation (164), and metabolic changes, including metabolic syndrome, are associated with the pathogenesis of osteoarthritis (146, 147). Additionally, the prevalence of osteoarthritis rapidly increases in women aged approximately 50 years, coinciding with menopause, suggesting a link between osteoarthritis and the protective effects of estrogen (106, 165). However, estrogen deficiency reduces BMSCs osteogenic potential and increases osteoclast formation, resulting in defective bone formation and osteoporosis (129). POSTN has also been reportedly involved in the osteogenic differentiation of BMSCs from ovariectomized rats through the estrogen-POSTN-Wnt/β-catenin pathway (11), and the association between estrogen and POSTN may also have some influence in AC degeneration.

Furthermore, the SASP actively secreted by SCs has been confirmed to lead to chronic inflammation, tissue degeneration, and, ultimately, osteoarthritis (152). However, some studies suggest that POSTN deposition causes chronic inflammation (166, 167), and POSTN activates NF-κB signaling and subsequently upregulates inflammatory cytokines and MMP expression in human chondrocytes (20). Moreover, POSTN deficiency prevents age-related spontaneous osteoarthritis, suggesting a link between POSTN and aging in the cartilage (61).

In several studies, serum and synovial fluid POSTN showed a significant positive correlation with radiographic severity in patients with knee osteoarthritis (85, 105). Additionally, lower serum POSTN levels have been linked with the prevalence of knee osteoarthritis and the risk of its development and progression in women (106). Therefore, these trends could be applied as biomarkers to determine the severity of knee osteoarthritis. However, the mechanism underlying the clearance of POSTN from the joint tissue to the serum is unknown (106), and further studies may be required to establish the clinical applicability of POSTN as a useful biomarker.

In the regeneration and tissue engineering of AC, which has made innovative progress recently, POSTN has been shown to enhance the mechanical strength of the surrounding fibrous tissue through structural changes in collagen molecules, activate extracellular signaling of cartilage cells by interstitial fibrous tissue, and contribute to maturation and shape retention of tissue-engineered cartilage (168). Thus, POSTN is also expected to be applied to cartilage regeneration therapy, which may lead to multifaceted osteoarthritis treatment.

## 6. POSTN is linked to intervertebral disk degeneration

### 6.1. Structure and function of IVD

The IVD contains three structures, including a central jelly-like NP, stiff outer annulus fibrosus (AF), and the upper and lower cartilaginous endplates, in addition to ECM components, all of which correlate with each other (169). AF is placed outside the circle of the IVD, surrounding the inner soft nucleus pulposus, whose function is to seal the nucleus and distribute the pressure and forces on the IVD, preventing herniation of the NP and leakage out of the disk (170). Resilient NP consists primarily of water (70%–90%), NPCs, proteoglycans, and type II collagen, which are important in supporting body weight and relieving pressure loads associated with spinal motion (171). The major components of the IVD, which are the outer AF and inner NP, synthesize ECM factors specific to cartilage (172).

Furthermore, damage to the NPC and AF, degeneration of the cartilage endplate, and calcification can occur as a result of various external and internal stimuli, impairing the normal biological function of the IVD and ultimately leading to IVDD.

Moreover, studies have shown that the onset and development of IVDD are triggered and accelerated by NPC depletion and are closely related to excessive catabolism of the ECM (173).

### 6.2. Impact of POSTN on IVDD

As POSTN is expressed predominantly in collagen-rich fibrous connective tissues that are subject to constant mechanical stress, the distribution of POSTN in the IVD is distinctive, with the outer annulus having the highest percentage of POSTN-positive cells (88.8%), followed by the inner annulus (61.4%) and the NPC (18.5%) (23). Notably, significantly increased expression of POSTN has been reported in the IVD of patients with IVDD (6, 25).

Histological examination revealed that human IVDs exhibit more fibrosis involving POSTN with structural disruption and fragmentation than non-degenerative IVDs (25). POSTN, which binds to ECM molecules in the IVD to initiate, maintain, and repair the IVDs (174).

Additionally, the development and onset of IVDD are closely related to excessive catabolism of the ECM, and studies have reported that *POSTN* may be a causative gene associated with IVDD development.

POSTN-mediated chondrocyte apoptosis and ECM degradation have been reported to promote cartilage degeneration and osteoarthritis (67); therefore, it can be inferred that a similar mechanism may be responsible for osteoarthritis changes in the cartilage component of the IVD.

### 6.3. POSTN-mediated effects of mechanical stress on IVDD

Evolutionarily, a two-legged upright posture is associated with a higher incidence of spinal lesions in humans than in four-legged mammals (175). Mechanical stress magnitude and duration positively correlate with the apoptosis rate of IVD cells, which is an important factor leading to IVDD and herniation through a complex mechanism (176). Additionally, mechanical stress increases POSTN and MMP2 expression, and the magnitude of the response in humans is significantly higher in degenerated NPCs than in non-degenerated cells (25). Therefore, POSTN expression stimulate or impede irregular collagen fiber formation and ECM organization to maintain or eliminate tissue homeostasis in response to mechanical stress. The expression of POSTN may promote or inhibit cell biological functions, stimulating or impeding irregular collagen fibrillogenesis and ECM organization to maintain or eliminate tissue homeostasis in response to mechanical stress and may induce chondrocyte apoptosis (61). Moreover, excessive loss of NP cells by apoptosis also disrupts ECM homeostasis and exacerbates IVDD progression (63).

### 6.4. POSTN-mediated effects of low-level inflammation on IVDD

Inflammation is a well-known and important feature of the IVDD environment (177). Spontaneous disk degeneration occurs when the NP, which is devoid of blood vessels, is exposed to circulation, resulting in inflammation and triggering an autoimmune inflammatory response (178). Notably, high levels of inflammatory cytokines, such as TNF-α, IL-1α/β, IL-6, and IL-17, secreted by disk cells have been postulated to induce IVDD (179, 180).

The expression levels of β-catenin, POSTN, and cleaved-caspase3 are significantly higher in severely degenerated IVDs than in mildly degenerated IVDs, and POSTN activates the Wnt/β-catenin pathway, which may promote apoptosis of NPCs and exacerbates degeneration (67).

However, the relationship between POSTN and inflammatory cytokines in IVDD cells remains poorly elucidated. Although there are limited studies on how POSTN and inflammatory cytokines lead to IVDD, crosstalk between POSTN and the most potent inflammatory cytokines, TNF-α and IL-1β, has been suggested as an underlying mechanism for various chronic inflammatory diseases. Furthermore, targeted therapies have been extensively studied in recent years, and POSTN-mediated regulation of myelinuclear cell apoptosis and inflammation, which are important pathological changes in IVDD, may be a promising targeted therapy approach in the future.

Therefore, the relationship between POSTN expression and IVDD has been elucidated, and potential mechanisms of NP cellular senescence have been studied on a genome-wide scale.

## 7. Lumbar spinal stenosis

LSS is one of the most severe issues among SDDs in older individuals owing to its high prevalence and negative impact on QOL (181, 182). LSS is defined as a syndrome characterized by the narrowing of the spinal canal, lateral recesses, and foramen nervosum, which are the pathways of the nervous system, causing specific symptoms in the lumbar region and lower extremities (181, 182). It is also caused by IVDD, facet osteoarthritis, and occasionally, vertebral fractures (183), resulting in intervertebral instability, compensatory thickening of the LF, and narrowing of the canals through which the nerves and the spinal cord pass. Moreover, inflammation-related scar mechanisms mediated by POSTN have been shown to cause the thickening of the lumbar LF (65).

Therefore, LSS is a combination of osteoporosis, disk degeneration, facet osteoarthritis, and thickening of the LF. Studies have shown that diabetes mellitus and metabolic syndrome, which are reportedly associated with POSTN (40, 41), are closely related to LSS (2–4).

Previous sections have explored the association of POSTN with facet osteoarthritis, IVDD, and osteoporotic vertebral fractures; this section will discuss the impact of POSTN on LF thickening in LSS.

### 7.1. Structure and function of LF

LF is a pair of ligaments that connect the ventral sides of adjacent vertebral arches. The lumbar spine is subjected to three-dimensional mechanical stresses, such as compressive, shear, and torsional forces. The remarkable elasticity of the yellow ligament serves to maintain an upright posture and to help the vertebrae recover their posture after flexion (65). Because the LF is posterior to the spinal canal, its degenerative hypertrophy can cause LSS (65, 184). Furthermore, LF hypertrophy progresses with age, and it is widely accepted that various factors, such as mechanical stress (185, 186), inflammation (187), and angiogenesis (188), contribute to LF hypertrophy.

Hypertrophy of the LF is an adaptive and reparative process that corresponds to the rupture of elastic fibers and is associated with an inflammatory fibrosis process involving POSTN (65). Additionally, pathology has shown severe fibrosis and degradation of elastic fibers in the thickened LF of the LSS (189).

### 7.2. Impact of POSTN on LF

Histologically, the LF comprises about 70% and 30% of elastin and collagen fibers, respectively (190). POSTN binds to multiple integrin molecules (αVβ1, αVβ3, αVβ5, α6β4, and αMβ2) in fibroblasts, which are largely involved in the biosynthesis of elastin fibers, the main component of this LF, and transmits activation signals into the cell (66).

The expression level of the *POSTN* gene in the enlarged LF tissue of the human LSS group was significantly higher than that in the LF tissue of the control group, indicating that the *POSTN* mRNA expression level is positively correlated with LF thickness (65). In hypertrophic LF, fibrosis-related factors, such as TGF-β1 (191), α-smooth muscle actin (α-SMA) (188), CoL1a1 (192), IL-6 (193), and MMP2 (189), are upregulated, and they reportedly promote the loss of elastic fibers during LF tissue degeneration. Because α-SMA and CoL1a1 are myofibroblast and ligament markers, respectively, it is logical to expect that these markers would be elevated in the thickened LF. TGF-β1 induces POSTN expression (66) and POSTN induces IL-6 and MMP2 via the FAK-NF-κB pathway (65), it can be speculated that POSTN plays a major role in the thickened LF.

### 7.3. POSTN-mediated effects of mechanical stress on the yellow ligament

Although mechanical stress is considered the primary factor involved in LF hypertrophy (194), the exact mechanism causing hypertrophy remains unclear. Concentrated mechanical stress has been reported to cause LF degeneration and hypertrophy in rabbits (195, 196). Yabu et al. (65) reported that degenerated and hypertrophied LF of rabbits showed increased collagen fibers, decreased density of elastic fibers, and disruption of fiber arrangement. TGF-β1 is significantly upregulated even in the hypertrophied LF (75, 191). In LF cells, mechanical stress increases TGF-β1 production (186), and TGF-β1 increases the synthesis of ECM proteins containing collagen (197). Furthermore, LF cells exposed to mechanical stress and TGF-β1 transdifferentiate from fibroblasts to myofibroblasts via the POSTN-integrin-NF-κB pathway, contributing significantly to LF hypertrophy (65).

Moreover, mechanical stress was found to cause abundant IL-6 expression involving fibroblasts in the LF of patients with LSS (185). IL-6 stimulated collagen expression in LF cells, leading to irreversible pathological LF remodeling, including fibrosis and loss of elastic fibers (198).

Therefore, mechanical stress can cause LF degeneration by inducing TGF-β1 expression, POSTN, and IL-6 via the NF-κB pathway.

### 7.4. POSTN-mediated effected of low-level inflammation on the yellow ligament

Sairyo et al. (187) reported that LF thickness positively correlates with the degree of fibrosis and that ligament thickening results from repeated inflammation, as scarring generally represents the final stage of the inflammatory process. POSTN derived from fibroblasts also acts on fibroblasts by collaborating with inflammatory cytokines, including TNF-α activating NF-κB, followed by inducing pro-inflammatory cytokines or chemokines (199). As mentioned above, mechanical stress causes POSTN to bind to integrin, inducing IL-6 and MMP via the FAK-NF-κB pathway and promoting inflammation and ligament degeneration (65). IL-6 is an important modulator of the inflammatory process involved in fibroblast differentiation, activation, and proliferation, and it affects ECM remodeling in several diseases (200). The JAK1/STAT3 pathway plays an essential role in the inflammatory response to thickening in the dorsal LF of LSS (201). Particularly, IL-6 activates JAK and STAT proteins (202, 203) and regulates MMP expression and activity. STAT3 is a transcription factor that induces MMP-2 expression and is believed to bind directly to the MMP-2 promoter in human tumor cells (204). Therefore, IL-6 may contribute to the degradation of elastic fibers by inducing MMP-2 through JAK/STAT3 signaling in LSS (189).

Furthermore, it has recently been proposed that age-related inflammation, or “infra-aging,” plays an important role in age-related disease development and progression (205). As POSTN reportedly plays a vital role in the cardiac vicious cycle (aging-fibrosis-inflammation-aging) by simultaneously promoting myocardial fibrosis and cardiac fibroblast aging (206), POSTN may also play a significant role in promoting inflammation through tissue aging.

Therefore, POSTN is a crucial molecule for LF hypertrophy and a promising therapeutic target for LSS.

## 8. Conclusion

Given the worldwide prevalence of SDDs, there is a critical need for effective disease-modifying treatment strategies to alleviate symptoms and slow SDD progression. POSTN levels are closely associated with the onset and progression of the diseases of the SDD component (osteoporotic vertebral fracture, facet osteoarthritis, IVDD, and LSS) resulting from both mechanical and inflammatory stress. Conversely, POSTN-mediated therapeutic interventions to modulate signals that amplify mechanical and inflammatory stress may restore physiological regulation and prevent SDD onset and progression. Some inflammatory diseases have provided insight into the stimulatory and inhibitory factors, expression levels, expression patterns, and roles of POSTN; signaling pathways involving POSTN; potential medical derivation; and utility as a promising marker in various diseases. Notably, diagnosis and severity determination of diseases using POSTN biomarkers and treatment with neutralizing antibodies have been practically applied in some inflammatory diseases. Therefore, POSTN may be a promising target for therapeutic strategies for SDDs. The development of new drugs for SDDs should focus on stimulators or inhibitors that affect POSTN expression, targeting POSTN itself, receptors or signaling pathways where POSTN is involved, or channels mediated by specific POSTN isoforms. Such approaches may have applicability as novel diagnostic and therapeutic targets in the treatment of SDDs.

## Author contributions

TY: Conceptualization, Investigation, Writing – original draft. TM: Conceptualization, Investigation, Supervision, Writing – original draft, Writing – review & editing, Data curation, Project administration. MMu: Investigation, Writing – original draft. TN: Investigation, Writing – original draft. MT: Data curation, Writing – review & editing. HH: Data curation, Writing – review & editing. YT: Data curation, Writing – review & editing. TK: Data curation, Writing – review & editing. KI: Conceptualization, Writing – review & editing. MMa: Data curation, Project administration, Supervision, Writing – review & editing.

## Glossary

AC: Articular cartilage
ACL: Anterior cruciate ligament
AF: Annulus fibrosus
AKC: Atopic keratoconjunctivitis
AS: Ankylosing spondylitis
CTD: Carboxy-terminal domain
ECM: Extracellular matrix
IVD: Intervertebral disk
IVDD: Intervertebral disk degeneration
LF: Ligamentum flavum
LSS: Lumbar spinal stenosis
MAPK: Mitogen-activated protein kinase
MMP: Matrix metalloproteinases
NP: Nucleus pulposus
NPC: Nucleus pulposus cells
OPLL: Ossification of the posterior longitudinal ligament
POSTN: Periostin
QOL: Quality of life
SASP: Senescence-associated secretory phenotype
SDD: Spinal degenerative disease
SCs: Senescent cells
TNF: Tumor necrosis factor
